# Humoral Response after a Fourth Dose with mRNA-1273 in Healthcare Workers with and without a History of SARS-CoV-2 Infection and Previously Vaccinated with Two Doses of BBIBP-CorV Plus BNT162b2 Vaccine

**DOI:** 10.3390/vaccines11050894

**Published:** 2023-04-25

**Authors:** Juan C. Gómez de la Torre, Miguel Hueda-Zavaleta, José Alonso Cáceres-DelAguila, Cecilia Muro-Rojo, Nathalia De La Cruz-Escurra, Vicente A. Benítes-Zapata

**Affiliations:** 1 Roe Clinical Laboratory, Dos de Mayo Avenue, 1741, San Isidro, Lima 15076, Peru; 2 Hospital III Daniel Alcides Carrión, Essalud, Calana Road, Km 6.5, Calana, Tacna 23000, Peru; 3 Facultad de Ciencias de la Salud, Universidad Privada de Tacna, Bolognesi Avenue Number 1177, Tacna 23003, Peru; 4 Unidad de Investigación para la Generación y Síntesis de Evidencias en Salud, Universidad San Ignacio de Loyola, La Fontana Avenue 550, La Molina, Lima 15024, Peru

**Keywords:** COVID-19, SARS-CoV-2, vaccine breakthrough infection, humoral immunity, heterologous booster vaccine, neutralizing antibody

## Abstract

There is limited information on the kinetics of the humoral response elicited by a fourth dose with a heterologous mRNA1273 booster in patients who previously received a third dose with BNT162b2 and two doses of BBIBP-CorV as the primary regimen. We conducted a prospective cohort study to assess the humoral response using Elecsys^®^ anti-SARS-CoV-2 S (anti-S-RBD) of 452 healthcare workers (HCWs) in a private laboratory in Lima, Peru at 21, 120, 210, and 300 days after a third dose with a BNT162b2 heterologous booster in HCW previously immunized with two doses of BBIBP-CorV, depending on whether or not they received a fourth dose with the mRNA1273 heterologous vaccine and on the history of previous SARS infection -CoV-2. Of the 452 HCWs, 204 (45.13%) were previously infected (PI) with SARS-CoV-2, and 215 (47.57%) received a fourth dose with a heterologous mRNA-1273 booster. A total of 100% of HCWs presented positive anti-S-RBD 300 days after the third dose. In HCWs receiving a fourth dose, GMTs 2.3 and 1.6 times higher than controls were observed 30 and 120 days after the fourth dose. No statistically significant differences in anti-S-RBD titers were observed in those HCWs PI and NPI during the follow-up period. We observed that HCWs who received a fourth dose with the mRNA1273 and those previously infected after the third dose with BNT162b2 (during the Omicron wave) presented higher anti-S-RBD titers (5734 and 3428 U/mL, respectively). Further studies are required to determine whether patients infected after the third dose need a fourth dose.

## 1. Introduction

More than 13 billion doses of vaccines against the Severe Acute Respiratory Syndrome Coronavirus 2 (SARS-CoV-2) virus have been administered worldwide [1], which have significantly reduced morbidity and mortality from the coronavirus disease 2019 (COVID-19) [2]. Currently, the World Health Organization (WHO) has authorized the emergency use of 11 vaccines against COVID-19 [3]. All these vaccines have proven effective and were used in different parts of the world according to availability. However, vaccines based on messenger ribonucleic acid (mRNA) platforms have shown greater efficacy and higher neutralizing antibody (NAbs) responses than other vaccine platforms, including inactivated vaccines [4]; also, it has been reported that heterologous vaccination schemes can potentiate a more significant immune response than homologous vaccination [5]. The level of neutralizing antibodies reflects the immunogenicity elicited by vaccines and has been used to predict the efficacy of vaccines against COVID-19 [6,7].

In February 2021, the Peruvian Ministry of Health authorized immunization against COVID-19 in healthcare workers (HCW), with two doses of the inactivated BBIBP-CorV vaccine (Beijing Bio-Institute of Biological Products Co Ltd., Beijing China), separated by 21 days [8]. Subsequently, before the advent of a third wave of COVID-19 due to the Omicron variant, in October 2021, the Peruvian Ministry of Health implemented a heterologous booster with the BNT162b2 vaccine (Pfizer-BioNTech, New York, NY, USA; Mainz, Germany) in HCW who had received a preliminary schedule of two doses of the inactivated BBIBP-CorV vaccine [9]. The third wave of COVID-19 in Peru occurred from January to March 2022, adding 1,135,010 cases of COVID-19. During this wave, the Omicron variant (B.1.1.529), which has shown high infectivity and immune evasion [10], was the main circulating variant isolated (99.3–100%), according to genomic sequencing data performed by the Peruvian National Institute of Health [11].

Given the rapid diffusion of the Omicron variant, added to the decrease in effectiveness and the level of NAbs after a third dose of the vaccine against COVID-19 [12]. On 2 April 2022, the Ministry of Health of Peru implemented a fourth dose of the mRNA1273 vaccine (Spikevax, Moderna Inc., Cambridge, MA, USA) against COVID-19 in HCWs previously immunized with three doses of the vaccine against COVID-19 [13].

There is limited information on the kinetics of the humoral response elicited by a fourth dose with a heterologous mRNA1273 booster in patients who previously received two doses of BBIBP-CorV and one dose of BNT162b2. Our study evaluated the humoral response in a quantifiable way, using the Elecsys^®^ anti-SARS-CoV-2 S assay at 21, 120, 210, and 300 days after a third dose with a BNT162b2 in HCW previously immunized with two doses of BBIBP-CorV, depending on whether or not they received a fourth dose with a heterologous mRNA1273 booster and according to the history of previous SARS-CoV-2 infection.

## 2. Materials and Methods

### 2.1. Study Design and Population

A prospective cohort study was conducted on a total of 452 healthcare workers (HCWs) in a private laboratory in Lima, Peru, who received a third dose of the COVID-19 vaccine with a heterologous booster of the BNT162b2 mRNA vaccine (Pfizer-BioNTech, New York, NY, USA; Mainz, Germany), seven months after two doses of the inactivated vaccine against SARS-CoV-2 (BBIBP-CorV), with or without a fourth dose with an mRNA-1276 booster vaccine. The study period was from November 2021 to October 2022. 

Before BBIBP-CorV vaccination, all blood samples from HCWs were evaluated monthly by the occupational health area. This area used two tests to detect IgM and/or IgG against SARS-CoV-2. One was the Aeskulisa SARS-CoV-2 S1 test (Aesku Diagnostics, Wendelsheim, Germany), an enzyme-linked immunosorbent assay (ELISA) against the S1 antigen. The other test was Elecsys^®^ Anti-SARS-CoV-2 (Roche Diagnostics International AG, Rotkreuz, Switzerland), a qualitative electrochemiluminescence immunoassay (ECLIA) that detects antibodies against the N antigen of SARS-CoV-2. Likewise, before, during, and after vaccination as part of the strategy to control the transmission of COVID-19 in HCWs, the occupational health area of the laboratory applied real-time polymer chain reaction (RT-PCR) or rapid antigenic test of SARS-CoV-2 in nasopharyngeal swabs in all HCWs that reported symptoms or contact with infected persons. This monitoring allowed us to distinguish the infected individuals before, during, and after vaccination. 

The HCWs were grouped into two cohorts at the time of anti-S-RBD dosing 300 days after the third dose with the BNT162b2 heterologous booster: (1) With previous SARS-CoV-2 infection (PI); (2) not previously infected with SARS-CoV-2 (NPI); according to the history of diagnosis of COVID-19 confirmed. All participants provided their written informed consent for this research. The study protocol was approved by the research ethics committee of the Private University of Tacna (protocol number 105/FACSA/UI, approved 15 July 2022).

### 2.2. Serological Tests

**Figure 1:**
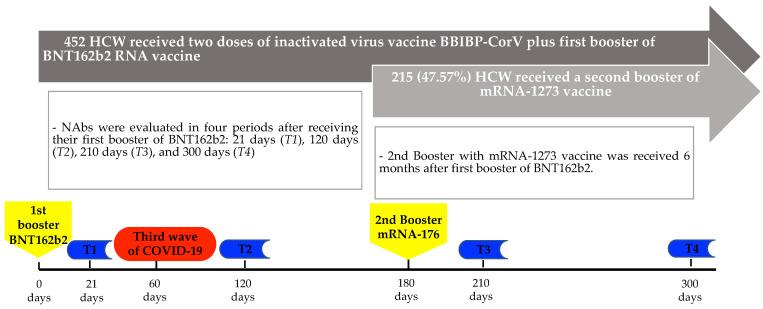
Timeline of the serological evaluations according to the vaccination period.

NAbs were measured at four moments: T1: 21 days, T2: 120 days, T3: 210 days, and T4: 300 days after receiving the third dose with BNT162b2 (Figure 1). The serological test used to quantify the humoral immunity against SARS-CoV-2 was Elecys^®^ anti-SARS-CoV-2 S (anti-S-RBD; Roche Diagnostics GmbH, Mannheim, Germany), which is a quantitative immunoassay that detects high-affinity total Ab against the receptor-binding domain (RBD) of the S protein of SARS-CoV-2, a value >0.8 U/mL was considered positive [14]. In the first and second monitoring (T1 and T2), only titers up to 2500 U/mL were determined. To improve the characterization of anti-S-RBD titers, we performed an additional dilution for the third and fourth monitoring (T3 and T4), which allowed us to determine titers up to 25,000 U/mL.

### 2.3. Statistical Analysis

Qualitative variables were reported as absolute and relative frequencies. Quantitative variables were reported as a median and interquartile range due to their non-normal distribution. Anti-S-RBD titers were reported as geometric mean (GMT) titers and 95% CI, as suggested by WHO guidelines [15]. The statistical analysis was performed using STATA V17.0 (StataCorp. College Station, TX, USA, EE.UU. 2019) and Prism V 9.2.0 (Graphpad Software, LLC, San Diego, CA, USA, EE. UU. 2021). Bivariate analysis was performed using chi2 or Fisher’s exact test for qualitative variables and U-Mann-Whitney or Wilcoxon signs and ranks, as appropriate. A value of *p* < 0.05 was considered statistically significant.

We performed a crude and adjusted linear regression analysis to investigate variables associated with a higher anti-S-RBD titer at 300 days after the third dose with a BNT162b2. Only those variables that met the assumptions of linearity, independence of observations, normality of residuals, and homoscedasticity were included in the analysis. Finally, to evaluate the variables associated with anti-SARS-CoV-2 S-RBD (anti-S-RBD) levels above the 75th percentile after 300 days of booster BNT162b2, we employed the Poisson regression model with robust variance to estimate the crude risk ratio (cRR) and the adjusted risk ratio (aRR) with a 95% confidence interval (95% CI)

## 3. Results

A total of 452 HCWs were included, of which 204 (45.13%) were previously infected. The median age was 34, and 347 (76.77%) were women. Most SARS-CoV-2 infections occurred before the first dose of BBIBP-CorV (52.45%) and after the third dose with BNT162b2 (44.60%). The median number of days from the first infection to the last control (T4) was 531. 47.57% (*n* = 215) of HCW received a fourth dose with a second heterologous booster with the mRNA1273 vaccine. No statistically significant differences were observed in any of these variables between previously infected (PI) and non-previously infected (NPI) HCWs (Table 1).

### 3.1. Elecys^®^ Anti-SARS-CoV-2 S (Anti-S-RBD)

**Figure 2:**
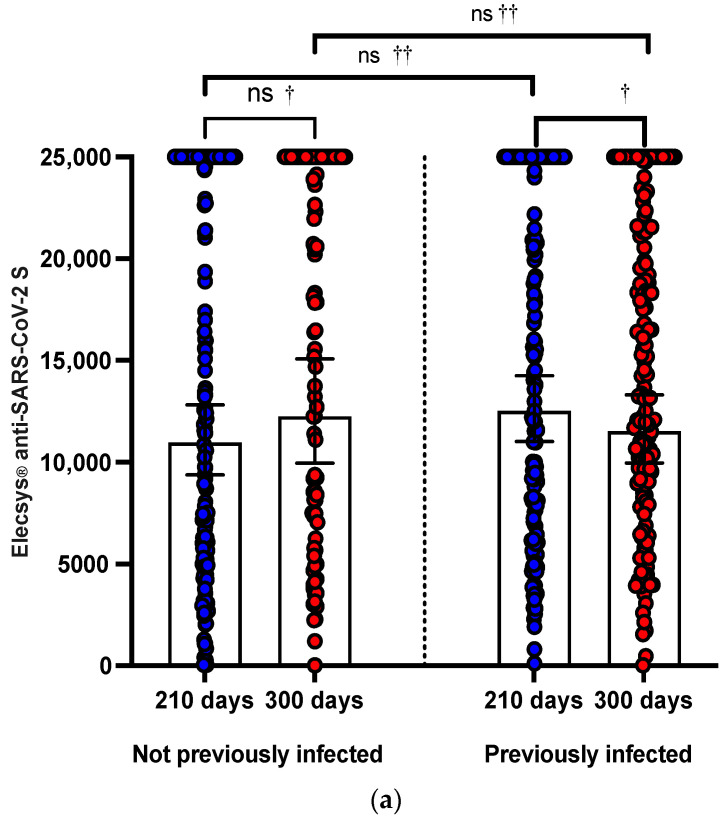

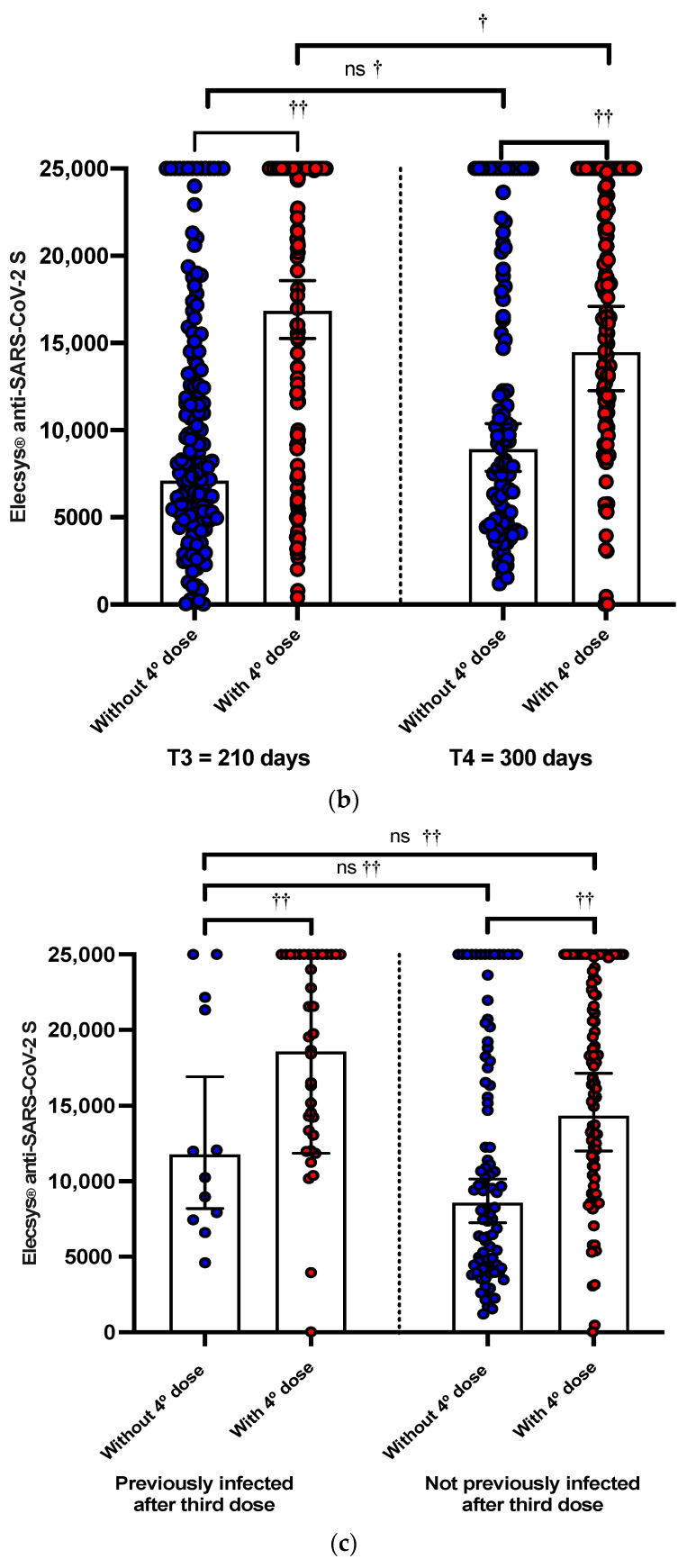
(**a**) The effect at 210 and 300 days of the third dose with booster BNT162b2 evaluated with Elecsys^®^ anti-SARS-CoV-2 S (anti-S-RBD). The boxplot shows GMT titers with 95% CI of an-ti-S-RBD antibodies determined by Elecsys^®^ Anti-SARS-CoV-2 S. Not statistically significant dif-ferences were observed in anti-S-RBD titers in HCW-NPI between 210 and 300 days (ns, †) but were in HCW-PI (†). Neither were differences observed between anti-S-RBD titers between HCW-NPI and PI at 210 and 310 days, respectively (ns, ††). (b) The effect at 210 and 300 days of the third dose with BNT162b2 evaluated with Elecsys^®^ anti-SARS-CoV-2 S. The boxplot shows the GMT titers with 95% CI of the NAbs determined by Elecsys^®^ Anti-SARS-CoV-2 S. These were significantly higher in those who received a second booster with the mRNA-1273 vaccine at 210 and 300 days after the third dose with BNT162b2, respectively (††). No differences were ob-served between anti-S-RBD titers in those who did not receive a fourth dose with mRNA-1273 at 210 and 300 days (ns, †), but yes, in those who received an mRNA-1273 booster (†). (c) The ef-fect at 300 days of the third dose with BNT162b2 according to the antecedent of infection after the third dose or not. The boxplot shows the GMT titers with 95% CI of the NAbs determined with Elecsys antiSARS-CoV-2 S. No differences were observed between anti-S-RBD titers in those who HCW were previously infected after the third dose, but that did not receive a fourth dose, and healthcare workers not previously infected after the third dose, but that did receive a fourth dose (ns, ††). ns, not significant; † Wilcoxon signed rank statis-tical test; †† U Mann–Whitney U test; GMT, geometric mean; 95% CI, 95% confidence intervals; NAbs, neutralizing antibodies; HCW, health workers; PI, previously infected; NPT, not previ-ously infected.

The proportion of patients with anti-S-RBD seropositive at 21 (T1), 120 (T2), 210 (T3), and 300 (T4) days after the heterologous booster with the BNT162b2 vaccine was 99.72%, 99.72%, 100%, and 100%, respectively. Only 01 HCW (0.28%) previously infected did not achieve anti-SARS-CoV-2 S seropositivity at 21 (T1) and 120 (T2) days. Anti-S-RBD titers increased significantly from 21 days to 120 days after receiving the third dose with the heterologous booster BNT162b2, from a GMT of 1.528 (95% CI: 1.281 to 1.822) to a GMT of 1.528. from 2381 (95% CI: 2269 to 2498; *p* < 0.0001) in HCW PI and from a GMT of 1406 (95% CI: 1152 to 1717) to a GMT of 2204 (95% CI: 1967 to 2470; *p* < 0.0001) at HCW NPI. Titers of anti-S-RBD titers at 210 (T3) and 300 (T4) days after the third dose with a booster BNT162b2 decreased significantly in GMTs of 12.526 (95% CI: 11.011 to 14,249) to a GMT of 11,512 (95% CI: 9957 to 13,310; *p* < 0.001) in HCW PI. No significant differences were observed in GMTs at 210 (T3) and 300 (T4) days after the third dose with a BNT162b2 heterologous booster in HCW NPI. (Figure 2a, Table 1)

### 3.2. Fourth Dose with mRNA1276 Heterologous Booster

In those HCWs who received a fourth dose with a heterologous mRNA1276 booster, we observed that the anti-S-RBD GMTs were 16,837.62 (95% CI: 15,254.7 to 18,584.79) and 14,476 (95% CI: 12,258.16 to 17,095.1) at 30 (T3) and 120 (T4) days after receiving the fourth dose, respectively. Meanwhile, in those who did not receive the fourth dose, GMTs were 7116 (95% CI: 5977.16 to 3428) and 8907.67 (95% CI: 7637 to 10,388.51) at T3 and T4, respectively (Figure 2b).

Finally, through adjusted linear regression, we identified that male HCWs presented 3810 IU/mL of anti-S-RBD higher than females. Similarly, those previously infected with SARS-CoV-2 after the third dose with a BNT162b2 booster showed 3428 IU/mL of anti-S-RBD higher than those who did not present this breakthrough infection. Likewise, HCWs who received a fourth dose of the mRNA-1276 vaccine showed 5734 IU/mL more elevated than those who only received a third dose with the BNT162b2 booster. In addition, it was observed that the HCWs PI before the third dose with the BNT162b2 vaccine presented lower antibody titers than their controls, although not statistically significant (Table 2). 

Moreover, the variables associated with anti-SARS-CoV-2 S-RBD (anti-S-RBD) levels above the 75th percentile after 300 days of booster BNT162b2, was the history of receiving a fourth dose with an mRNA-1273 booster that was associated with anti-S-RBD titers above the 75th percentile. In contrast, a history of infection before the primary regimen with BBIBP-CorV was associated with a lower possibility of presenting anti-SARS-CoV-2 S-RBD anti-S-RBD titers above the 75th percentile (Table 3).

## 4. Discussions

We observed that 100% of HCW were anti-S-RBD positive at seven months after a third dose with a heterologous BNT162b2 booster, regardless of whether they were previously infected or received a fourth dose with a second mRNA-1276 heterologous booster. Furthermore, anti-S-RBD levels increased significantly after administering a fourth dose with mRNA1276 and in those who suffered breakthrough COVID-19 infection after receiving the third dose with BNT162b2 during the third wave of COVID-19 in Peru. Omicron and sub-variants BA.4 and BA.5 were the predominant circulating variants during this period [11].

As in other studies [16], we observed that the increase in anti-S-RBD after a fourth dose was higher than that observed after a third dose [17]. The COV-BOOST clinical trial evaluated the immunogenicity of a fourth dose with BNT162b2 or mRNA1273 after two doses of ChAdOx1 nCoV-19 or BNT162b2 and a third dose of BNT162b2, observing that the increase in GMT was higher with mRNA1273 than with BNT162b2 (2.19 versus 1.59 times the baseline) [16]. In addition, a study in Israel showed that a fourth dose with mRNA1273 caused higher NAbs and anti-RBD levels over time than BNT162b2 [18]. This finding coincided with a slightly higher vaccine efficacy against infection and symptomatic disease with mRNA1273 than BNT162b2 [18,19].

Although the overall history of previous SARS-CoV-2 infection did not impact anti-S-RBD titers, when the infection occurred after the third dose (during the Omicron wave), it was associated with a higher anti-S-RBD titer. This finding is consistent with other studies [20], and it is presumed that this hybrid immunity (vaccination plus natural infection) has an improved humoral and cellular response due to a greater number of B cells, neutralizing antibodies, and differentiated CD4 T cells that produce Interferon-gamma and interleukin-10 [21]. However, the increase in anti-S-RBD caused by a natural infection in our cohort was lower than that caused by a fourth dose with the second booster with mRNA1273, which suggests a higher immunogenicity caused by vaccination.

The previous exposure to SARS-CoV-2 can hypothetically reduce the risk of reinfection and its severity [22], the emergence of variants such as Omicron with immune evasion capacity [23] and the fading of this protection over time [24], make previously infected patients vulnerable to reinfection. This raises concern due to the findings reported by Bowe B. et al., where reinfection increased the risk of death, hospitalization, and acute and long-term sequelae in the re-infected patients’ pulmonary, cardiovascular, hematologic, and neurological systems. Likewise, the cumulative risks increased according to the number of infections [25]. Therefore, until more evidence is available in this regard, we believe that previously infected patients, regardless of their humoral response, should be immunized in the same way as those not previously infected.

It has been reported that after the fourth dose, a peak of antibodies occurs at four weeks and subsequently decreases progressively between 11 to 14% of GMT weekly, reaching the levels observed before the fourth dose at 13 weeks and stabilizing after that during a 6-month follow-up period [26]. In our study, a decrease in anti-S-RBDs was also observed in those who received a fourth dose with a second mRNA1273 booster at 3-month follow-up.

Regarding the need for a fourth dose, we must mention that although the third dose can induce antibodies against Omicron, these titers were lower than other variants such as alpha, beta, and delta [27]. Since there is no cut-off point to indicate the level of seroprotective antibodies against COVID-19, it is unknown if the NAbs induced by the third dose are sufficient or if a fourth dose is required. It has been observed that after a third dose with BNT162b2, NAbs levels reach a peak 30 days after the booster and have a half-life of 44 to 58 days, so it can be estimated that it would take approximately 255–326 days for antibody levels to decrease to pre-booster levels [28]. We must also consider that the T-cell response is essential to confer protection against severe disease by COVID-19 [29], and this cellular response seems to be more long-lived than the humoral response [30]; it may be stimulated after exposure to Omicron in individuals previously infected or vaccinated [31], and the T-cell response was similar after a fourth or third dose against COVID-19 [16]. 

Surprisingly, no decrease in anti-S-RBDs was observed during follow-up in those who received only a third dose with the heterologous booster BNT162b2, even up to 300 days after the third dose, which is inconsistent with other authors [32]. We cannot rule out that this effect is secondary to many breakthrough infections during the third wave of COVID-19, which was associated with a higher anti-RBD titer as a reflection of a hybrid immune response.

It has been observed that after a fourth dose of mRNA1273 or BNT162b2, there is a 9–10-fold increase in the anti-RBD titer [33]. Despite this, initially, no benefit was observed against SARS-CoV-2 infection when applying a fourth dose of mRNA1273 or BNT162b2 in HCW [34], but later, studies with larger populations have reported protection (RR: 0.35; 95% CI: 0.32–0.39) against breakthrough infection COVID-19 [35].

Currently, there is no NAbs breakpoint indicating protection against SARS-CoV-2 infection. There is an association between immunogenicity and protection against COVID-19. For example, a study showed that an anti-spike IgG level below 350 BAU/mL was associated with an increased risk of COVID-19 during the wave of Omicron [36]. 

Since SARS-CoV-2 is an RNA virus, generating mutations in its genome is to be expected. Most of these mutations fail to alter their structure, but some can alter their sequencing and the composition of amino acids in essential proteins, which could give rise to new variants. Currently, multiple variants are circulating worldwide, many with mutations that decrease the effectiveness of vaccines against COVID-19, even with high NAbs titers [37,38,39]. The emergence of these variants has motivated the use of bivalent vaccines, which the Food and Drug Administration (FDA) has approved for the immunization of patients older than six months in the case of the Moderna vaccine and patients older than five years with the Pfizer-BioNTech vaccine [40]. These vaccines have mRNA from the ancestral strain of SARS-CoV-2 and the BA.4 and BA.5 variants and were designed to counteract the immunological evasion of the Omicron subvariants compared to the monovalent vaccine, and have shown safety, even in children from 5 to 11 years [41]. However, no statistically significant differences were observed in NAbs levels against BA.4 and BA.5 variants, nor in CD4+/CD8+ T-cell responses after receiving a monovalent or bivalent booster [42,43]. It is likely that the immune systems of people immunized with the bivalent vaccine were primed to respond to epitopes from the ancestral SARS-CoV-2 strain and, probably when exposed to the bivalent vaccine, responded to these epitopes shared by BA.4, BA.5, and the SARS-CoV-2 strain ancestral, rather than new epitopes of BA.4 and BA.5 [44]. Even so, the effectiveness data of a bivalent mRNA vaccine against hospitalization due to COVID-19 have recently been published, being 73% compared to people who received two or more doses of monovalent mRNA vaccine and 91% in those who did not were immunized [14]. 

Despite the availability of vaccines against COVID-19, lower coverage of booster doses has been observed in the population globally, even among health personnel. Strategies are needed that promote booster vaccination regardless of age, type of vaccine received, or history of previous infection. In addition, it is necessary to improve confidence in vaccines and the facility to access them [45].

To our knowledge, no studies have evaluated the humoral response in people immunized with a combination of mRNA-1276, BNT162b2, and BBIBP-CorV vaccines. However, our study has some limitations. First, most HCWs were young and without comorbidities, so we cannot extrapolate our findings to the general population. Likewise, it was impossible to specify NAbs against the main circulating concern variants, such as Omicron, which was predominant during the study period. Moreover, the additional dilution performed in our study is likely to be problematic in all laboratories due to requiring an extra processing step. It is possible that some patients have suffered asymptomatic infections and have not been diagnosed during study follow-up after vaccination since it was not possible to continue monitoring with serological methods as a result of immunization with BBIBP- CorV; this is relevant due to the high prevalence of asymptomatic SARS-CoV-2 infections (between 20 to 40%); it is still not very clear if asymptomatic people produce a humoral response similar to symptomatic people [46,47,48]. Finally, we did not have a control group that received the two heterologous boosters with BNT162b2 or two homologous BBIBP-CorV boosters to compare the levels of NAbs between them, and due to the study’s observational nature, and without randomized sampling, we cannot rule out the presence of some selection bias. Controlled clinical trials are required in the future.

## 5. Conclusions

The anti-S-RBD levels increased significantly after administering a fourth dose with mRNA1276 and in those who suffered breakthrough COVID-19 infection after receiving the third dose with BNT162b2 during the third wave of COVID-19 in Peru. All authors have read and agreed to the published version of the manuscript.

## Figures and Tables

**Table 1:** Demographic characteristics and humoral response rates of the study population and comparison between healthcare workers previously infected and not previously infected.

Variable	Total (*n* = 452)	Previously Infected (*n* = 204)	Not Previously Infected (*n* = 248)	*p*-Value
Demographic characteristics				
Age, years *	34.0 (28–43)	34 (29–43)	33 (28–41)	0.251 ^a^
Age categorized (*n* = 424) (%)				0.479 ^b^
- 18–34 years	227 (53.53)	102 (44.93)	125 (55.07)	
- 35–49 years	145 (34.20)	68 (46.90)	77 (53.10)	
- 49–64 years	47 (11.09)	25 (53.19)	22 (46.81)	
- ≥65 years	5 (1.18)	1 (20.00)	4 (80.00)	
Sex (%)				0.613 ^b^
- Female	347 (76.77)	159 (45.82)	188 (54.18)	
- Male	105 (23.23)	45 (42.86)	60 (57.14)	
Previously infected (%)				
- Before the first dose of BBIBP-CorV	107 (52.45)	107 (100.0)	-	-
- After the second dose of BBIBP-CorV	36 (17.64)	36 (100.0)	-	-
- After the third dose with BNT162b2	91 (44.60)	91 (100.0)	-	-
Days from the first infection *	531 (251–582)	531 (251–582)	-	-
Fourth dose with mRNA-1273 (%)	215 (47.57)	102 (47.44)	113 (52.56)	0.347 ^b^
Humoral response after the third dose with BNT162b2 by Elecsys anti-SARS-CoV-2 S (U/mL).				
- T1: Titles at 21 days (*n* = 358) **	1467 (1285–1674)	1528 (1281–1822)	1406 (1152–1717)	0.618 ^a^
- T2: Titles at 120 days (*n* = 358) **	2284 (2140–2437)	2381 (2269–2498)	2204 (1967–2470)	0.289 ^a^
- T3: Titles at 210 days (*n* = 350) **	11,640 (10,497–12,908)	12,526 (11,011–14,249)	11,966 (9382–12,817)	0.269 ^a^
- T4: Titles at 300 days (*n* = 237) **	11,794 (10,468–13,287)	11,512 (9957–13,310)	12,243 (9945–15,071)	0.026 ^a^

^a^: U Mann-Whitney; ^b^: Chi2; * Median (interquartile range); ** Geometric means and 95% confidence interval.

**Table 2:** Simple and multiple linear regression of the variables associated with titles of antibodies anti-SARS-CoV-2 S-RBD (anti-S-RBD) after 300 days of third dose with BNT162b2.

Elecys Antibodies Anti-SARS-CoV-2 S (Anti-S-RBD) after the Third Dose with BNT162b2 at 300 Days (T4)				
Variable	Coeficiente β Crudo (95% IC)	Valor *p*	Coeficiente β Ajustado (95% IC)	Valor *p*
Male	2632.93 (23.83–5242.02)	0.048	3810 (1405.76–6216.00)	0.002
Age categorized				
- 18–34 years	Reference	-	-	-
- 35–49 years	493.42 (−1832.87–2819.72)	0.676	-	-
- 50–64 years	3015.02 (−71.95–6102.00)	0.056	-	-
- ≥65 years	6481.60 (−590.21–13,553.42)	0.072	-	-
Previously infected	−1869.57 (−3950.80–211.64)	0.078	−2571.84 (−5489.68–345.98)	0.084
- Before the first dose of BBIBP-CorV	−3000.78 (−5150.77–−850.80)	0.006	−1252.70 (−3908.30–1402.89)	0.354
- After the second dose of BBIBP-CorV	−3961.05 (−7449.32–−472.77)	0.026	−2088.93 (−5525.41–1347.55)	0.232
- After the third dose with BNT162b2	2916.77 (437.62–5395.92)	0.021	3428.08 (638.12–6218.04)	0.016
Fourth dose with booster mRNA-1273	5981.33 (4057.88–7904.78)	0.000	5734.63 (3838.54–7630.73)	0.000

95% IC: 95% confidence interval, SARS-CoV-2: Severe Acute Respiratory Syndrome Coronavirus 2; BBIBP-CorV: Inactivated vaccine against SARS-CoV-2 BBIBP-CorV; BNT162b2: Vaccine ARNm BNT162b2; mRNA-1273: mRNA-1273 SARS-CoV-2 vaccine; Reference: the reference category used to calculate the B coefficients of the other categories.

**Table 3:** Simple and multiple Poisson regression of the variables associated with titers of antibodies anti-SARS-CoV-2 S-RBD (anti-S-RBD) above the 75th percentile after 300 days of third dose with BNT162b2.

Variable	cRR (95% CI)	*p*-Value	aRR (95% CI)	*p*-Value
Male	1.462 (0.900–2.374)	0.125	-	-
Age categorized				
- 18–34 years	Reference	-	Reference	-
- 35–49 years	1.022 (0.590–1.772)	0.936	1.009 (0.608–1.674)	0.972
- 50–64 years	1.843 (1.074–3.164)	0.026	1.639 (0.970–2.769)	0.065
- 65 years	2.723 (1.231–6.023)	0.013	2.537 (0.825–7.803)	0.104
Previously infected	0.430 (0.275–0.673)	0.000	0.642 (0.389–1.059)	0.083
- Before the first dose of BBIBP-CorV	0.319 (0.159–0.640)	0.001	0.427 (0.201–0.909)	0.027
- After the second dose of BBIBP-CorV	0.336 (0.088–1.290)	0.112	-	-
- After the third dose with BNT162b2	1.360 (0.841–2.199)	0.210	-	-
Fourth dose with booster mRNA-1273	2.007 (1.203–3.349)	0.008	2.206 (1.311–3. 711)	0.003

cRR: crude risk ratio, aRR: adjusted risk ratio, 95% IC: 95% confidence interval, SARS-CoV-2: Severe Acute Respiratory Syndrome Coronavirus 2; BBIBP-CorV: Inactivated vaccine against SARS-CoV-2 BBIBP-CorV (Sinopharm); BNT162b2: Vaccine ARNm BNT162b2 (Pfizer/BioNTech); mRNA-1273: mRNA-1273 SARS-CoV-2 vaccine (Moderna). Reference: reference category used to calculate the B coefficients of the other categories.

## Data Availability

The data analyzed in this manuscript, as well as its definitions, can be downloaded at the DOI.
